# Mass Spectrometry Imaging: Revolutionizing Molecular Insights in Infectious Diseases Research

**DOI:** 10.3390/pathogens14070645

**Published:** 2025-06-30

**Authors:** Minmin Zhang, Xiao Wang, Xiaoling Su, Aidiya Yimamu, Lanjuan Li, Zeyu Sun

**Affiliations:** 1 Jinan Microecological Biomedicine Shandong Laboratory, Jinan 250117, China; 2 State Key Laboratory for Diagnosis and Treatment of Infectious Diseases, National Clinical Research Center for Infectious Diseases, Collaborative Innovation Center for Diagnosis and Treatment of Infectious Disease, The First Affiliated Hospital, School of Medicine, Zhejiang University, Hangzhou 310003, China

**Keywords:** infectious diseases, mass spectrometry imaging, microorganism, biomarkers, pharmacology

## Abstract

Infectious diseases remain a leading cause of mortality worldwide. The pathogenesis that comprises infection, focal inflammation, and immuno-response, typically occurs in one or multiple organs or tissues. Analysis of the molecular composition of affected tissues with their spatial context is pivotal to elucidate the underlying disease mechanisms and to develop accurate diagnostic strategies. In recent years, mass spectrometry imaging (MSI) technology has achieved significant advancements and has emerged as an powerful tool for tissue-based molecular exploration with high molecular specificity and spatial resolution. Although MSI has been rapidly adopted in numerous branches of biomedical research, its application in the field of infectious diseases research is still in its early stages. With this in mind, this review aims to familiarize infectious disease researchers with the advantages and diverse applications of MSI. Additionally, we delineate several existing technical challenges, application pitfalls, and the potential solutions to overcome these challenges.

## 1. Introduction

Infectious diseases of viral, bacterial, fungal, and parasitic origin, pose serious threats to human health and impose substantial socioeconomic burdens. It is predicted that global mortality related to infectious diseases could reach 10 million by 2050 [1]. Histologically, infectious diseases evolve from pathogen invasion through sequential phases of innate and adaptive immune activation and tissue microenvironment restructuring, accompanied by substantial localized molecular signature shifts [2,3]. Conversely, key molecular features such as antigens, xenobiotic toxins, pathogen-associated molecular patterns (PAMPs)/damage-associated molecular patterns (DAMPs), or even host metabolites, can ultimately shape the outcome of infectious disease [4,5,6]. Immunohistochemical staining (IHC) [7], immunofluorescence imaging, and fluorescence in situ hybridization (FISH) [8] are common techniques to visualize molecular profiles in tissues. However, these methods are labor-intensive and time-consuming. Moreover, antibody-based or hybridization-based methods can only be applied to limited targets, mostly peptides, proteins, or nucleic acids, with limited multiplexing capabilities [9]. Visualizing the distribution of chemical compounds such as toxins and metabolites is particularly challenging using these conventional approaches. This limitation is now being addressed by mass spectrometry (MS)-based omics techniques, such as metabolomics, lipidomics, and proteomics, which facilitate high-throughput biomarker screening [10] and elucidate infection mechanisms [11,12]. Recently, it has transformed both clinical management and research paradigms in the field of infectious diseases. The most prominent examples are the rapid microbial identification and single nucleotide polymorphism (SNP) genotyping techniques using matrix-assisted laser desorption/ionization mass spectrometry (MALDI-MS) with high sensitivity, accuracy, and high throughput [13]. However, MS-based assays typically rely on isolation or extraction of targeted analytes *prior* to profiling without spatial information. To address these shortcomings, in situ MS techniques have been introduced to directly map the chemical composition on tissue or culture samples [14]. This newly emerging tool, namely mass spectrometry imaging (MSI), is a label-free, three-dimensional (3D) method and has seen rapid technological progress, enabling its widespread adoption in diverse biomedical fields, including oncology [15,16], neurology [17], cardiovascular diseases [18], liver or gastric diseases, and more [19].

Nevertheless, the potential of MSI for infectious disease research remains largely untapped. This review will highlight MSI-based studies related to infectious disease, while critically analyzing current technical limitations and proposing potential solutions. With this comprehensive review, we hope to inspire and enable researchers to expand its use in investigating infectious pathogens.

### Mass Spectrometry Imaging: Concepts and Workflows

The MSI technique was first proposed by Caprioli et al. in 1997 [20,21], to simultaneously determine the mass, abundance, and spatial distribution of chemical compounds. The MSI data acquisition workflow involves the following: (1) partitioning the sample surface into discrete pixel regions, (2) detecting chemicals via their *m*/*z* values using the mass analyzer, (3) correlating these measurements with precise X and Y coordinates, and (4) reconstructing the spatial chemical composition for each pixel. Upon scanning the entire target region, a spatial distribution image of all detected compounds is drawn.

**Figure 1:**
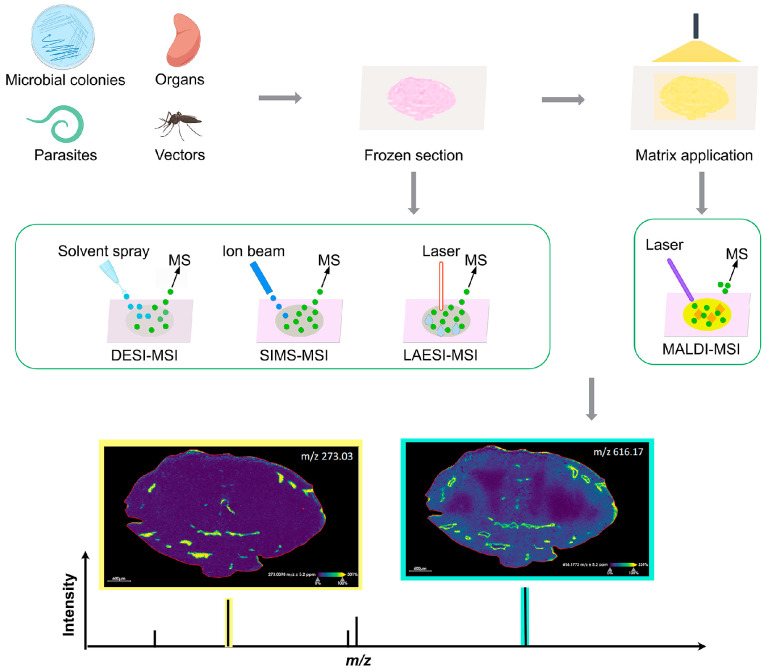
Brief flowchart of mass spectrometry imaging (MSI) with a frozen section of tissue. The sample is taken, snap-frozen, sectioned, and mounted onto a glass slide for MSI analysis with a proper ionization technique. For matrix-assisted laser desorption/ionization (MALDI)-MSI, an additional step of matrix application is also needed.

To perform MSI analysis, molecules must first be ionized directly on the targeted sample surface. Common ionization techniques include matrix-assisted laser desorption/ionization (MALDI), desorption electrospray ionization (DESI), secondary ion mass spectrometry (SIMS), and laser ablation electrospray ionization (LAESI). MALDI-MSI, which accounts for the majority of MSI publications, offers distinct advantages for biomolecule analysis [22]. The technique accommodates various sample types ranging from organs (e.g., spleen, liver, brain, and skin), to smaller biological samples (parasites, vectors, and even microbial colonies). As a classical process, samples are frozen in the embedding medium and then sectioned into dimensions of less than 1 × 1 cm and a thickness of about 8–15 µm [23]. Fresh frozen tissue specimens are preferred to minimize unwanted oxidation or degradation during long-term storage, thereby to preserve spatial organization. However, MSI can be adapted to explore formalin-fixed paraffin-embedded (FFPE) samples, as discussed later. The cut specimen is then mounted onto a conductive slide or plate, dried by vacuum, and coated with matrix to assist ionization [24]. With the help of matrix molecules, MALDI-MSI creates charged ions of analytes from tissue surfaces by scanning through planar slices with a pulsed laser. These ions are then simultaneously identified by MS with great sensitivity and selectivity. Visualization of the spatial distribution of each detected analyte is achieved by extracting the mass intensity profile from all spatially resolved MS scans across the entire section of tissue under investigation [25] (Figure 1).

As summarized in Table 1, each ionization source has certain pros and cons. MALDI-MSI can visualize a wide range of molecules, including small chemical compounds (<500 Da, e.g., drugs or metabolites), peptides, and even large proteins (up to 70 kDa). Nevertheless, when used to visualize small molecules, it is susceptible to background interference from matrix molecules. Continuous efforts have been made to introduce new matrices and derivative methods to customize MALDI-MSI for diverse sample types and targeted analytes [26]. Currently, the introduction of post-ionization with a second laser (MALDI-2) has drastically increased signal intensities for many classes of small molecule compounds and has brought MALDI-MSI analysis to the subcellular level with pixel sizes below 1 µm [27].

Secondary ion mass spectrometric imaging (SIMS-MSI) offers the highest spatial resolution to visualize finer details. While SIMS ionization is matrix-free, the sample slide needs to be dry and vacuum-stable, similar to MALDI-MSI [28,29]. In contrast, both LAESI-MSI and DESI-MSI, which operate under ambient pressure, are compatible with hydrated biofilms and do not require desiccation or matrix assistance. Notably, LAESI achieves 5 µm spatial resolution through laser focusing via lens systems or chemically etched/microlensed fibers [30]. In this case, samples must contain water to resonate with the laser to sputter and evaporate compounds, which are captured and ionized by the electrospray. DESI-MSI exhibits exceptional sample versatility by directly dispersing electrically charged droplets onto the tissue surface. While this method primarily targets small molecules with a spatial resolution of 50~200 µm, its nanospray variant (nanoDESI) achieves a resolution of 10 µm [31], enabling single-cell DESI analysis.

Following ionization, the charged analytes can be detected by various types of mass analyzers with different sensitivity, mass resolution, and dynamic range. In a typical MSI experiment, full ion spectra (MS1) are collected first to record the *m*/*z* values and ion intensities of the analytes across the scanned region. These analytes can be further selected as precursors and fragmented to obtain tandem mass spectra (MS2) that contain the *m*/*z* values and ion intensities of structurally informative fragment ions derived from the precursor. MALDI-MSI and SIMS-MSI are commonly coupled with a time-of-flight (TOF) mass analyzer operating on the principle that ions with different *m*/*z* ratios traverse a uniform accelerating electric field at varying speeds and durations [32]. Higher resolution mass analyzers, such as a Fourier transform ion cyclotron resonance (FT-ICR) analyzer or an Orbitrap analyzer, provide more robust and specific analyses. Specifically, the Orbitrap analyzer [33], which works by radially trapping ions around a central spindle electrode, is increasingly favored for its high-mass resolution to identify biomolecules. Recently, ion mobility spectrometry (IMS), which separates ions based on their size or collision cross-section (CCS), has been integrated into MS instruments to provide an additional separation dimension for MSI analysis, making it particularly effective in distinguishing isomeric compounds.

The combination of ionization techniques and the variety of high-performance mass analyzers has resulted in numbers of MSI systems capable of investigating molecular composition, distribution, and dynamics across tissue and even cellular scales [34] and exploring unknown biochemical species inaccessible to other conventional methodologies [35].

## 2. Application of MSI in Infectious Diseases

### 2.1. Identify Biomarkers of Infectious Disease Progression

In precision medicine, biomarkers are crucial for early screening and diagnosis, evaluating disease progression and prognosis, as well as for monitoring therapeutic response and adverse effects. Infection induces distinct spatial alterations in tissue distribution and the abundance of proteins and small molecules that may serve as potential biomarkers. The discovery of novel biomarkers essentially relies on comprehensive molecular profiling in different biological states or processes, necessitating large-scale detection of molecules to begin with. Unlike traditional approaches, MSI enables simultaneous spatial mapping of both known markers and uncharacterized molecular patterns directly within diseased lesions (Table 2).

Pu et al. used an airflow-assisted ionization (AFAI) MSI system to compare the spatial distribution of metabolites in liver tissues from healthy individuals and patients with hepatitis B virus (HBV)-related liver cirrhosis (LC) [36]. Alterations in several classes of metabolites were found to correlate with LC progression, with significant decreases in arginine, proline, phosphatidylcholines (PCs), lysoPCs, and fatty acid species. These spatially resolved molecular perturbations can indicate region-specific autophagic activity and may contribute to the identification of novel topographic biomarkers for LC staging. Granulomatous inflammation—a pathological hallmark of leishmaniasis—is associated with changes in both host and parasite lipid metabolism, as observed at the bulk tissue level across multiple infection models [37]. In a descriptive study using MALDI-MSI to define spatial-temporal lipidomic changes in mouse livers infected by *Leishmania donovani*, 34 histology-registered lipids displayed markedly spatial heterogeneity between granulomas and parenchyma [38]. The MSI can be used in parallel with other molecular imaging techniques to identify disease biomarkers. For example, Matys et al. successfully applied Fourier transform infrared (FT-IR) spectroscopy and MALDI-MSI to probe chemical composition modulated by infection of opportunistic bacteria *Aeromonas* sp. FT-IR spectra revealed an overall biochemical ‘fingerprint’ including proteins and lipids that can discriminate healthy tissue from infected tissue, whereas MALDI-MSI unveiled the spatial-temporally resolved lipidomic landscape, particularly lysoPCs dysregulation, and provided a reliable molecule profile of tissues during the infection process [39]. Fincher et al. developed a platform called silicon nanopost arrays (NAPA) for laser desorption ionization (LDI) to visualize neutral lipid species such as triglycerides (TGs) [40]. They showed that the TG levels were related to increased bacterial load in the skin tissue, which was simultaneously confirmed by scanning electron microscopy (SEM). This study also identified hexosylceramides (HexCers) and galabiosyl-/lactosylceramides as potential biomarkers for hidradenitis suppurativa (HS) infection, which leads to chronic and recurrent apocrine sweat gland inflammation.

**Table 2:** Summary of infectious disease biomarkers identified by MSI technology.

Reference	Infectious Disease	Technology	Biomarker
[36]	Hepatitis B virus (HBV)-related liver cirrhosis (LC)	Airflow-assisted ionization (AFAI) MSI	Arginine, proline, phosphatidylcholines (PCs), lysoPCs, and fatty acids
[38]	Leishmaniasis	MALDI-MSI	34 histology-registered lipids, such as phosphatidylglycerols (PGs)
[39]	*Aeromonas* infection in kidney tissue	Fourier transform infrared (FT-IR) spectroscopy and MALDI-MSI	I/amide II or CH_2_/CH_3_ absorbance index, Lysophosphatidylcholine (LPC), PCs, and diacylglycerol (DAG)
[40]	Hidradenitis suppurativa (HS)	Laser desorption ionization (LDI) MSI and scanning electron microscopy (SEM)	Hexosylceramides (HexCers) and galabiosyl-/lactosylceramides
[41]	*Staphylococcus aureus*-induced infective endocarditis (IE)	Laser ablation inductively coupled plasma (LA-ICP) MSI	Calcium, magnesium, and zinc

In addition to biomolecules, infection triggers distinct metallic ion signatures, offering new avenues for diagnostic marker development. Schwarz and colleagues used laser ablation inductively coupled plasma (LA-ICP) MSI to generate distribution maps of metal elements in murine heart valves with *Staphylococcus aureus*-induced infective endocarditis (IE) and verified that an increased level of calcium, magnesium, and zinc could indicate the presence of *S. aureus*, which can be distinguished from sterile inflammation [41]. Notably, the presented approach can be used as a standard tool to identify infected herds through characteristic metallic signatures.

### 2.2. Capturing Host-Pathogen Interactions

Infectious diseases start with the invasion and reproduction of pathogens, including bacteria, viruses, fungi, protozoa, and parasites, followed by subsequent host response to pathogens or their toxins [42]. Elucidating the infection mechanism requires understanding chemical interactions within microbial cultures [43,44,45] and host–microbe interfaces [46,47]. In one example, chemical distributions throughout sagittal planes of biofilms forms by *Bacillus subtilis* sub-species were acquired by 3D MALDI-MSI and correlated with the 3D distributions of *B. subtilis* phenotypic reporters visualized by confocal laser scanning fluorescence microscopy in the biofilm periphery [48]. In turn, the authors pinpointed the signaling role of surfactin for extracellular matrix formation and revealed the critical role of iron-scavenging bacillibactin for the bacterium’s survival within complex microbial consortia.

**Figure 2:**
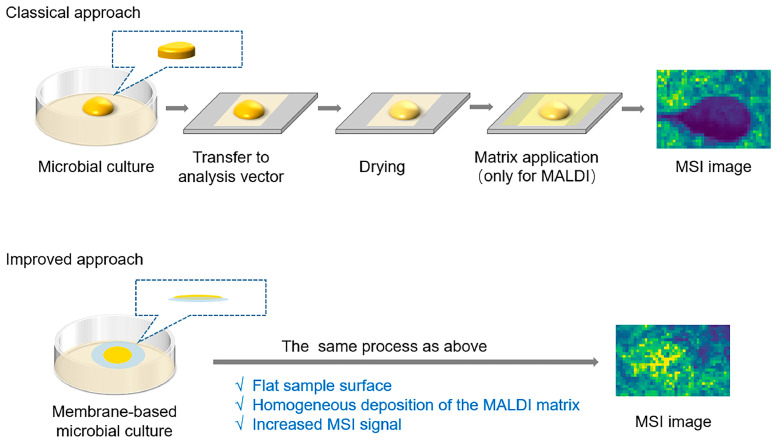
The preparation process of microbial culture for MSI. In the conventional approach, microbial culture is directly incubated on agar, followed by transfer to analysis vector, drying, and matrix application prior to the MSI analysis. In the improved approach, a membrane placed between the microorganisms and the culture medium allows spores to readily spread out on the surface of the membrane, which improves both the homogeneous deposition of the MALDI matrix and the mapping of molecules.

MSI is a convenient tool to inquire into chemical interactions within multi-species microbial communities. Frydenlund Michelsen et al. used MALDI-MSI to analyze agar cocultures of *Pseudomonas aeruginosa* and *Staphylococcus aureus*, and revealed that the signal metabolite 4-hydroxy-2-alkylquinoline released by *P. aeruginosa* can protect *S. aureus* from a tobramycin challenge, demonstrating interspecies cooperation during polymicrobial infection [49]. Clearly, metabolites are the mediators underlying the basic processes of recognition, communication, and manipulation across interactions. In addition to the above sample preparation method for microbial colonies, Slimani et al. developed a polymeric cellophane membrane-based technique for MALDI-MSI analysis of anomalous filamentous bacterial colonies like *Streptomyces ambofaciens* (Figure 2). Using this method, they mapped the spatial distribution of *S. ambofaciens*-produced metabolites like desferrioxamine E in an agar medium Furthermore, kinetic investigations of bacteria metabolites on agar media were made possible by quantitative analysis of an MS signal [50]. In another example, MALDI-MSI was used to distinguish various natural vs. cultured *Trichodesmium* morphotypes based on spatial differences in metabolite abundance. In conjunction with liquid extraction surface analysis (LESA) that collects MS2 fragmentation spectra of significant varying metabolites, Romanowicz et al. identified 80 distinct compounds associated with single-colony *Trichodesmium* morphotypes [51].

MSI can also visualize chemical interactions between hosts and parasites or symbiotes. The Carbapenem-resistant *Klebsiella pneumoniae* sequence type 258 (Kp ST258) is a major cause of healthcare-associated pneumonia. Despite producing immunostimulatory lipopolysaccharide, Kp ST258 often evades bacterial clearance, resulting in protracted courses of infection. To explore the possible mechanism, Wong et al. combined in situ DESI-MSI metabolome mapping with transcriptional analyses to define immune cell populations in murine lungs [52]. The MSI results revealed that Kp ST258 activated host glutaminolysis and fatty acid oxidation, creating an oxidant-rich microenvironment conducive to the accumulation of anti-inflammatory myeloid cells and M2-like macrophages, therefore eliciting immune tolerance. The bacteria also upregulated the type VI secretion system in response to airway oxidants and adapted to the oxidant-replete milieu.

*Rhabditis maupasi* resides as a parasite in the nephridia and coelomic cavities of common earthworm species. Geier et al. utilized MALDI-MSI and synchrotron radiation-based micro-computed X-ray tomography (SR-micro-CT) to gain insights into metabolic interactions between the earthworm host and its parasites [53]. In this work, SR-micro-CT revealed distinct histopathological states of the nematode and a humoral response (e.g., a platelet activation factor lipid concentrating at the host–parasite interface) of the earthworm against the nematodes in the coelomic cavity. Meanwhile, the detailed metabolic profile obtained by atmospheric pressure (AP)-MALDI-Orbitrap-MSI showed that the spermidine, as an anti-oxidative compound could help the nematodes to survive until the earthworm sheds its posterior segments, providing an escape mechanism from their host. These studies and many others illustrate the potential of MSI for analyzing microbial monocultures in liquid or agar media as well as complex systems when combined with other multimodal techniques.

Known as nutritional immunity, the host secretes metalloproteins to sequester nutrient metals to prevent microbial colonization. Opportunistic pathogens such as *S. aureus* employ siderophores (e.g., staphyloferrins) to scavenge Fe, bypassing host nutritional immunity defenses. In one example, MALDI-FT-ICR-MSI revealed that *S. aureus* siderophores staphyloferrin A and B were concentrated at infection sites in the heart, liver, and kidney tissues of mice, and their location correlated with depleted Fe visualized by laser ablation inductively coupled plasma (LA-ICP) MSI. Such competition for metal between the host and pathogen revealed by MSI was further confirmed by H and E staining and fluorescence microscopy [54]. These results provide insight into microbial metal acquisition during infection and emphasize the capabilities of MSI to investigate host-microbe interactions.

### 2.3. Visualizing Therapeutic Mechanisms

Imaging analysis by MSI integrates molecular imaging with the histological context and enhances comprehensive spatial pharmacokinetics and the pharmacodynamic (PK/PD) profiling of therapeutic agents within tissue microenvironments. In particular, precision therapy requires that drug molecules reach the lesion region at a sufficient unbound concentration to ensure pharmacological efficacy. Plasma concentration has traditionally been used as a surrogate in PK/PD studies, but it cannot accurately elucidate drug distribution in specific lesion substructures. To fill this gap, drug distribution has traditionally been assessed by whole-body autoradiography and microautoradiography, which require expensive and laborious radioactive isotopes for tracking. In comparison, MSI can directly detect unlabeled molecules along with their metabolites in a single experiment, eliminating bottlenecks associated with the synthesis and use of radiolabeled compounds. As an example, traditional pharmacological methods have difficulties in assessing antiretroviral drug (ARV) levels in morphologically heterogeneous tissues, such as lymph nodes (LNs), which serve as reservoirs for persistent HIV infection. Rosen et al. utilized multimodal imaging approaches to quantify spatial relationships between drug and targets (blood, virus, and T cells), namely, RNAscope in situ hybridization for viral RNA (vRNA) and IHC for T cell and collagen expression. Through MSI, heterogeneous distribution of multiple ARVs was observed in different morphological LN regions, such as the follicles and medullary sinuses of rhesus macaques infected with a reverse transcriptase-SIV expressing HIV-1 envelope (RT-SHIV). The spatial mapping of drug, immune cells, and vRNA provided contextual insights into the impact of the tissue microenvironment, information that would be lost by tissue homogenization or by analyzing isolated cells [55]. In MSI, its flexible framework allows ARVs to be evaluated individually or collectively and offers a tool to optimize the PK/PD of HIV therapy.

It is known that infection sites often form intricate microenvironments with varying degrees of inflammation, necrosis, and tissue or vascular remodeling, all of which markedly affect antibiotic permeability. Antibiotics that penetrate these sites cannot be elucidated using conventional PK/PD techniques, whereas in situ MSI can also be used to optimize dosing regimens and to reduce drug resistance development. This was demonstrated by a recent MSI study for the PK/PD evaluation of anti-tuberculosis (TB) drugs [56], revealing that rifampicin and pyrazinamide can effectively penetrate TB lesions, while moxifloxacin and clofazimine, despite their high anti-TB activity in vitro, implicate limited mouse efficacy and clinical utility, as suggested by other studies [57,58]. By using chloroform as a tissue solvent to wash sections prior to matrix application, it was possible to detect these antibiotics using MALDI-FT-ICR-MSI scanning of lung sections from mice with TB infection with high reproducibility. Subsequent research used this technique to investigate the lesion PK of amikacin, kanamycin, and vancomycin in TB-infected lung granulomas and bone tissue [59]. Altogether, this protocol enhanced MSI sensitivity by significantly removing endogenous ion suppressants and cleared the way for investigations into antibiotic penetration at the site of disease.

A key advantage of MSI is its ability to map drugs and their metabolites simultaneously. This was illustrated by Gunawardana and co-workers, who used MALDI-MSI to characterize the spatial co-distribution of tenofovir alafenamide (TAF) and its five principal metabolites in the surrounding tissues of a subdermal implant for HIV prophylaxis [60]. The extremely labile substance TAF and its metabolites were typically found near the implant, except on day 28 when dispersion increased, while tenofovir (TFV) and its phosphorylated prodrug metabolites exhibited uniform tissue distribution with varying concentrations. These data established the effective dosage of TAF to prevent vaginal and rectal acquisition of HIV and addressed knowledge gaps in the preclinical pharmacology of long-acting subdermal TAF delivery.

Moreover, MSI serves as a transformative tool in drug development, enabling direct visualization of novel therapeutics and delivery systems within tissues to validate targeting efficacy and biodistribution. *Schistosoma mansoni* is a parasitic flatworm that causes schistosomiasis, which affects several hundred million people worldwide. Praziquantel (PZQ) stands as the sole therapeutic agent for schistosomiasis treatment, but its monotherapeutic use raises significant concerns about potential resistance emergence. Given PZQ’s resistance risks, drug repurposing efforts identified the anti-cancer imatinib as a promising alternative candidate. Mokosch et al. applied high-resolution AP scanning microprobe MALDI-MSI (AP-SMALDI-MSI) to investigate cryosections of imatinib treated *S. mansoni* [61] and revealed that imatinib was absorbed both orally and via the tegumental surface of the worms, as well as tropism towards the ovary. The parallel detection of its main metabolite, N-desmethyl imatinib, suggested metabolization of the drug by the multicellular parasite. In another multimodal imaging study, the distribution of the antibacterial medication GSK2485680, administered as a liposomal formulation (Lipo680), was assessed in a mouse thigh model of bacterial infection [62]. MALDI-MSI was used to quantify the distribution of GSK2485680 and to qualitatively assess the distribution of liposomal lipids throughout sections of infected and non-infected hindlimb tissues at high spatial resolution. In parallel, positron emission tomography (PET) imaging showed the selective accumulation of Lipo680 at the infection site. These multimodal data reveal excellent correlations and help to reduce drug attrition by generating comprehensive biodistribution profiles to validate targeted delivery and formulation integrity.

Collectively, these studies demonstrate that MSI is a powerful analytical tool to provide comprehensive drug distribution data early in drug discovery and development, as well as insights into local biochemical changes that are directly related to drug transport and activity, further elucidating the various mechanisms behind antibiotic resistance in order to develop new treatment strategies.

## 3. Aspects Worth Considering 

To facilitate the broader application of MSI in basic research and clinical management of infectious diseases, several critical technical challenges must be addressed.

### 3.1. Formalin-Fixed Paraffin-Embedded Tissues

While fresh frozen (FF) specimens are preferred for MSI-based experiments, FFPE samples constitute the majority of archived pathological specimens that are accompanied with valuable clinical records and metadata.

Most formalin-fixed tissue sections are fragile and have low adherence to the glass slide. Moreover, paraffin embedding could cause a significant washout of polar small molecules, including metabolites, lipids, and pharmaceutical compounds, as well as macromolecule cross-linking. The breakdown of materials in tissues and the possibility of artifacts may be influenced by exposure sampling, intricate processing stages, sluggish fixation kinetics, paraffin removal, tissue washing techniques, and residual enzyme activity. Valid data gathering from these tissue slices depends on effective sample preparation.

**Figure 3:**
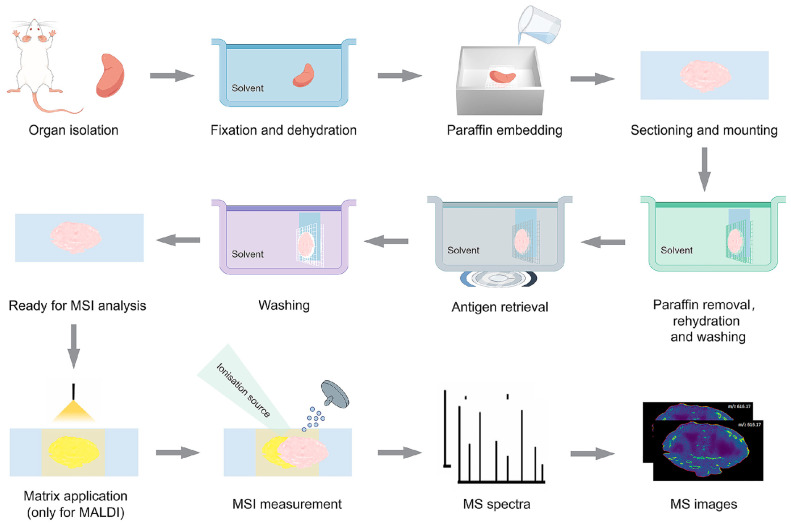
Experimental workflow for the MSI analysis of formalin-fixed paraffin-embedded (FFPE) samples. The organs are rinsed with paraformaldehyde (PFA) solution before being isolated, fixed, dehydrated, and embedded in paraffin with a standard procedure. Before MSI analysis, paraffin removal, antigen retrieval, or tryptic digest are then performed; meanwhile, washing steps are interspersed to facilitate the tissues to be ready for matrix application.

Recent reports highlight that MSI has been successfully adapted for molecule characterization. Hermann and co-workers developed a protocol for MALDI-MSI to detect peptides in various FFPE tissue types, with high sensitivity, spatial resolution, and reproducibility [63], as summarized in Figure 3. The incorporation of a mild antigen retrieval step appeared to enhance MALDI-MSI detection of solvent-resistant lipids in human FFPE renal samples [64]. Meanwhile, continuous improvements in MSI mass resolution could simplify sample preparation and improve the detection of low-abundance metabolites commonly masked by dominant background signals in FFPE samples. Buck et al. pioneered the in situ MALDI-FT-ICR-MSI investigation of metabolites (e.g., hexose-6-phosphate, and glycerol) from FFPE tissue samples prepared only by deparaffinization and matrix application [65]. A comparison between FFPE and FF tissue metabolome over a mass range of *m*/*z* 50–1000 revealed analogous metabolite spatial profiles with a 72% overlap of detected compounds. However, molecules in the *m*/*z* 50–400 range were more abundant in the FFPE sample, while signals in the *m*/*z* 600–1000 range were more prominent in the FF sample. They concluded that sample preparation had minimal impact on peak detection, confirming the capability of MALDI-FT-ICR-MSI for measuring metabolites in FFPE tissues. Moreover, a novel platform known as AP infrared laser-ablation plasma post-ionization (AP-IR-LA-PPI) was developed to identify ions derived from a variety of hydrophilic small molecular metabolites (such as succinate and adenine) and lipid classes from FFPE kidney tissues of mice without post-sectioning sample preparation [66]. This promising new ion source allows FFPE and other “incompatible” sample forms to be more readily analyzed by MSI.

### 3.2. Highly Infectious Samples

In infectious disease studies, special attention must be given to samples that necessitate strict adherence to biosafety requirements for processing and examination. Preparation of tissue sections for MSI analysis may generate infectious aerosols, posing potential contamination risks. While it is technically possible to set up an MSI platform together with auxiliary instruments within BSL-3/4 laboratories, such arrangements are rare in practice due to high operational costs. Therefore, specimens with high infectivity need to be inactivated prior to MSI analysis. Cazares et al. designed a study to investigate Venezuelan equine encephalitis virus (VEEV) and *Burkholderia*-infected tissues using MSI. Heat fixed tissue for pathogen inactivation produced a pattern similar to that of fresh frozen tissue [67], indicating that the treatment had no effect on the distribution or content of analytes and that heat fixation inactivated bacterial and viral pathogens while maintaining tissue stability and compatibility for MALDI-MSI analysis of protein/peptide, lipid, and small molecule analytes. However, heat treatment could potentially affect tissue morphology and compromise the detection of small molecules. Dannhorn et al. tested ultraviolet-C (UV-C) radiation as an alternative decontamination technique in their DESI-MSI study of cryo-sectioned tissues infected with various pathogens. including herpesviridae, papovaviridae, human immunodeficiency virus, and SARS-CoV-2 [68]. They concluded that a 3 h decontamination cycle at high doses of UV-C, certified for high-level decontamination, caused oxidation and photodegradation of endogenous species, whereas a 30 min decontamination cycle employing low doses of UV-C radiation effectively inactivated pathogens while preserving the tissue metabolome and xenobiotics relatively intact. This protocol minimizes the risk of unwanted contamination from clinical specimens prior to MSI analysis, thereby improving operational safety and broadening the utility of MSI in infectious disease research.

## 4. Conclusions

Although further technological refinements are needed, MSI represents a transformative approach for tissue-based investigations in microbiology, pharmacology, and clinical diagnostics. By providing targeted or untargeted highly multiplexed molecular imaging data, MSI emerges as an compelling alternative tool that not only complements but also could potentially supersede conventional chemical- or antibody-based imaging techniques. This technique enables unprecedented visualization of metabolic exchange between microorganisms and their hosts across various sample matrices, including microbial cultures to histological slices, in both 2D and 3D spatial formats. Given the ongoing development of innovative MSI methodologies for biomedical applications, this technology is poised to revolutionize infectious disease research in the foreseeable future.

## Figures and Tables

**Table 1:** Characteristics and important metrics of common MSI techniques.

Parameter	Matrix-Assisted Laser Desorption/Ionization (MALDI)	Desorption Electrospray Ionization (DESI)	Secondary Ion Mass Spectrometry (SIMS)	Laser Ablation Electrospray Ionization (LAESI)
Sample	Fresh frozen tissue/formalin-fixed paraffin embedding	Fresh frozen tissue	Fresh frozen tissue/single cell	Fresh frozen tissue
Required sample preparation	Matrix application	No	Freeze fracture and drying for subcellular imaging	Hydrous
Ionization conditions	Atmospheric/medium/high vacuum	Atmospheric vacuum	Ultrahigh vacuum	Atmospheric vacuum
Mass range	10–100,000	100–10,000	1–3000	1–5000
Spatial resolution	5–200 µm	10–200 µm	0.05–100 µm	5–300 µm
Probing depth	0.1–20 µm	1–500 µm	0.001–10 µm	40–400 µm
Coupled analyzer	TOF/Q-TOF/FT-ICR/Orbitrap	Q-TOF/FT-ICR/Orbitrap	TOF/Q-TOF/FT-ICR	TOF/FT-ICR/Orbitrap
Destructive	Minor	Minor	Significant	Significant
Usage rate	Far beyond the average	Above the average	On average	Below average

## Abbreviations

The following abbreviations are used in this manuscript:

MSI	Mass spectrometry imaging
PAMPs	Pathogen-associated molecular patterns
DAMPs	Damage-associated molecular patterns
IHC	Immunohistochemical staining
FISH	Fluorescence in situ hybridization
MS	Mass spectrometry
SNP	Single nucleotide polymorphism
MALDI	Matrix-assisted laser desorption/ionization
3D	Three-dimensional
DESI	Desorption electrospray ionization
SIMS	Secondary ion mass spectrometry
LAESI	Laser ablation electrospray ionization
FFPE	Formalin-fixed paraffin-embedded
SIMS-MSI	Secondary ion mass spectrometric imaging
TOF	Time-of-flight
FT-ICR	Fourier transform ion cyclotron resonance
IMS	Ion mobility spectrometry
CCS	Collision cross-section
AFAI	Airflow-assisted ionization
HBV	Hepatitis B virus
LC	Liver cirrhosis
PCs	Phosphatidylcholines
PGs	Phosphatidylglycerols
FT-IR	Fourier transform infrared
NAPA	Nanopost arrays
LPC	Lysophosphatidylcholine
DAG	Diacylglycerol
HS	Hidradenitis suppurativa
LDI	Laser desorption ionization
TGs	Triglycerides
SEM	Scanning electron microscopy
HexCers	Hexosylceramides
LA-ICP	Laser ablation inductively coupled plasma
IE	Infective endocarditis
LESA	Liquid extraction surface analysis
Kp ST258	*Klebsiella pneumoniae* sequence type 258
AP	Atmospheric pressure
SR-micro-CT	Synchrotron radiation-based micro-computed X-ray tomography
PK	Pharmacokinetics
PD	Pharmacodynamics
ARVs	Antiretroviral drugs
LNs	Lymph nodes
vRNA	Viral RNA
RT-SHIV	Reverse transcriptase-SIV expressing HIV-1 envelope
TB	Tuberculosis
TAF	Tenofovir alafenamide
TFV	Tenofovir
PZQ	Praziquantel
AP-SMALDI-MSI	AP scanning microprobe MALDI-MSI
PET	Positron emission tomography
FF	Fresh frozen
AP-IR-LA-PPI	AP infrared laser-ablation plasma post-ionization
PFA	Paraformaldehyde
VEEV	Venezuelan equine encephalitis virus
UV-C	Ultraviolet-C
